# Risk of influenza infection with low vaccine effectiveness: the role of avoidance behaviour

**DOI:** 10.1017/S0950268818003540

**Published:** 2019-01-25

**Authors:** Thomas N. Vilches, Majid Jaberi-Douraki, Seyed M. Moghadas

**Affiliations:** 1 Department of Biostatistics, Institute of Biosciences, São Paulo State University, Botucatu SP 18618-689, Brazil; 2 Department of Mathematics, and Department of Anatomy and Physiology, Institute of Computational Comparative Medicine, Kansas State University, Manhattan, KS 66506, USA; 3 Agent-Based modelling Laboratory, York University, Toronto, Ontario M3J 1P3, Canada

**Keywords:** Agent-based simulation model, avoidance behaviour, influenza, risk-of-infection, vaccine effectiveness

## Abstract

Low vaccine-effectiveness has been recognised as a key factor undermining efforts to improve strategies and uptake of seasonal influenza vaccination. Aiming to prevent disease transmission, vaccination may influence the perceived risk-of-infection and, therefore, alter the individual-level behavioural responses, such as the avoidance of contact with infectious cases. We asked how the avoidance behaviour of vaccinated individuals changes disease dynamics, and specifically the epidemic size, in the context of imperfect vaccination. For this purpose, we developed an agent-based simulation model, and parameterised it with published estimates and relevant databases for population demographics and agent characteristics. Encapsulating an age-stratified structure, we evaluated the per-contact risk-of-infection and estimated the epidemic size. Our results show that vaccination could lead to a larger epidemic size if the level of avoidance behaviour in vaccinated individuals reduces below that of susceptible individuals. Furthermore, the risk-of-infection in vaccinated individuals, which follows the pattern of age-dependent frailty index of the population, increases for older age groups, and may reach, or even exceed, the risk-of-infection in susceptible individuals. Our findings indicate that low engagement in avoidance behaviour can potentially offset the benefits of vaccination even for vaccines with high effectiveness. While highlighting the protective effects of vaccination, seasonal influenza immunisation programmes should enhance strategies to promote avoidance behaviour despite being vaccinated.

## Background

Vaccination against seasonal influenza remains a primary public health measure to prevent infection and its outcomes such as hospitalisation and death [1, 2]. The impact of this measure, however, depends on several factors including vaccine coverage and more importantly vaccine effectiveness [3]. The effectiveness of vaccines produced by the conventional egg-based method is subject to virus mutation that can occur both during the production process [4], and during natural infection [5]. The occurrence of these mutations during seasonal epidemics enhances the antigenic distance between dominant influenza viruses and the vaccine strains [4], further reducing vaccine effectiveness over the course of an epidemic [6], especially in high-risk individuals and those with a high frailty index [7]. Frailty is defined as state of increased vulnerability and reduced functioning, and is often considered as a predictive measure of health outcomes.

Although higher vaccine coverage can, to a limited extent, compensate for low vaccine effectiveness, the risk-of-infection also depends on other individual-level characteristics such as behavioural responses [8]. For example, contact avoidance can have a significant impact on reducing disease transmission [9–11]. However, the perceived risk-of-infection is an important factor in practicing such behavioural responses [12], and intervention measures that reduce the risk-of-infection (e.g. vaccination) may also reduce the level of avoidance behaviour among target groups. In simple terms, since vaccination aims to protect individuals, the perceived lower risk-of-infection in vaccinated individuals may influence their level of engagement in avoidance behaviour compared to susceptible individuals [13]. In rare cases of fully protective vaccines, such behavioural changes in vaccinated individuals can be disregarded in disease spread. However, influenza vaccination often has low effectiveness [3, 9, 14–16], especially in the geriatric population and individuals with high frailty [5]. If vaccination offers less incentive to forgo beneficial but potentially infectious contacts, then the epidemic size may increase despite lower susceptibility conferred by the vaccine-induced protection. We sought to investigate and quantify the risk-of-infection in the context of vaccination and avoidance behaviour. This quantification can help vaccination campaigns to improve communication on the effect of influenza vaccine and the role of other measures including avoidance behaviour to impede disease transmission.

To achieve the objective of this study, we developed an agent-based simulation model for the spread of influenza, and parameterised it with available data and estimates from previous studies. We analysed the outcomes in the context of mixing patterns of individuals in order to measure the variation in the risk-of-infection in different age groups.

## Methods

### The model framework

We developed a stochastic age-structured agent-based model of influenza transmission dynamics, which includes epidemiological statuses of susceptible (*S*), latent (*L*), symptomatic infection (*I*), asymptomatic infection (*A*) and recovered (*R*). Recovery from infection was assumed to confer immunity against re-infection in the same epidemic season.

### Population study and contact patterns

Demographics of the population were sampled from census data collected by Statistics Canada (Fig. S1, Supplementary Material) [17]. Due to short timelines of a single influenza epidemic season, we ignored demographics of birth and natural death. We considered 15 age groups for the population and used the average of contact matrices in POLYMOD study for European countries as a proxy for matrix of contact patterns in urban structures [18]. The daily number of contacts for each individual was sampled from an age-specific negative-binomial distribution (Table S1, Supplementary Material) and randomly assigned to individuals in different age groups (Fig. S2, Supplementary Material).

### Disease dynamics

Disease transmission occurred through contacts between susceptible and symptomatically or asymptomatically infectious individuals, and was implemented as rejection sampling-based (Bernoulli) trials where the chance of success is defined by a transmission probability distribution [19, 20]. The baseline transmission probability was obtained by calibrating the model to a specific reproduction number (*R*_0_ = 1.4) within the estimates for influenza outbreaks [21].

Following exposure to infection and disease transmission, the newly infected individuals enter the latent stage during which the disease cannot be transmitted. Once the latent period has elapsed, the disease may manifest as symptomatic or asymptomatic infection. Durations of latent and infectious periods were sampled for each individual from the associated distributions described in the Parameterisation section.

### Vaccine effectiveness

Vaccination was implemented randomly to achieve the vaccine coverage reported for seasonal influenza in different age groups [22]. For a given vaccine efficacy *V*_e_ (defined as infection prevention in randomised control trials involving healthy individuals [5]), the protection level of a vaccinated individual against infection was determined by *E* = *V*_e_(1 − *f*), where *f* represents the frailty index. We considered *E* as the individual-level vaccine effectiveness. We sampled the frailty index for each individual by performing a segmented linear regression as a function of age (Fig. S4, Supplementary Information), fitted to the 2014 Canadian Community Health Survey data of chronic diseases [23]. The vaccine effectiveness was included as a reduction factor for disease transmission. This effectiveness also reduced the probability of developing symptomatic infection by a factor of 1 − *E* in vaccinated individuals if infection occurred. The transmission probability per contact was then obtained by
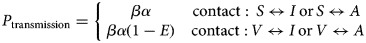
where *α* represents the level of infectiousness, with *α* = 1 for symptomatic infection (*I*), and *α* < 1 for asymptomatic infection (*A*).

### Behavioural responses

We modelled behavioural responses of individuals using a parameter *p*_s_ (*p*_v_), representing the fraction of contacts that are avoided by susceptible (vaccinated) individuals with symptomatically infectious individuals. The effect of changes in contact patterns during the epidemic has been investigated in previous studies [24, 25]. We assumed that vaccinated individuals have a lower contact avoidance compared to susceptible individuals, and therefore considered *p*_v_ ⩽ *p*_s_. This assumption corresponds to a higher number of contacts post-vaccination. Although quantifying human behaviour is challenging, a previous study reports that in the 2 days immediately following influenza vaccination, individuals socially encountered almost twice as many contacts as they did in the 2 days prior to vaccination [13].

### Per-contact risk-of-infection

To estimate the risk-of-infection in each age group throughout the epidemic, we used a maximum-likelihood approach. Since infection per contact was implemented as a Bernoulli trial, we defined a likelihood function for the risk in susceptible individuals by (assuming the independence of events):
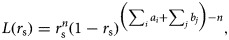
where *n* is the number of infections generated by contacts between susceptible and infectious individuals; 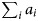 is the total number of contacts that led to infection as the outcome and 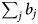 is the total number of contacts between susceptible and infectious individuals without infection as the outcome. For each scenario, we used the maximum likelihood estimator:
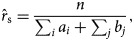
in independent realisations to determine the risk in different age groups. Since vaccine-induced protection reduced the probability of infection, we considered a parameter *c* as the reduction factor for the per-contact risk-of-infection (*r*_v_) in vaccinated individuals and defined the likelihood function by:
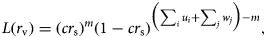
where *m* is the number of infections generated by contacts between vaccinated and infectious individuals; 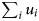 is the total number of contacts that led to infection as the outcome and 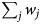 is the total number of contacts between vaccinated and infectious individuals without infection as the outcome. Given 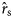, we used the likelihood estimator:
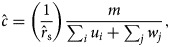
to determine the per-contact risk-of-infection among vaccinated individuals in different age groups.

### Parameterisation

We parameterised the simulation model with a total population size *N* = 10 000 individuals, with a transmission probability that was calibrated to the reproduction number *R*_0_ = 1.4 in the absence of any control measures [21]. A recent systematic review estimates the median *R*_0_ = 1.28 for seasonal epidemics, with a range of 1.11–2.2 [21]. The reproduction number, which was calculated by introducing an individual in the latent state of disease in independent realisations and averaging the number of new symptomatic cases generated, reflects the epidemic growth at the early stages and changes during the course of the epidemic (Figs S8 and S9, Supplementary Material). Latent period was drawn from a uniform distribution with the mean of 1.5 days within the estimated ranges [26, 27]. The infectious periods for both symptomatic and asymptomatic infections were sampled from a truncated lognormal distribution with scale parameter *μ* = 1 day and shape parameter *σ*^2^ = 0.4356 (Fig. S3, Supplementary Material), having a mean of 3.38 days [27, 28]. The probability of developing asymptomatic infection was sampled for each individual from a uniform distribution in the range 0.3–0.7 [27]. We assumed that the infectiousness of asymptomatic infection is reduced by 50% compared to symptomatic infection [27]. Vaccine efficacy (*V*_e_) was varied between 0.2 and 0.8, from which the vaccine effectiveness (*E*) for each vaccinated individual was calculated based on the sampled frailty index (Fig. S5, Supplementary Material). Vaccine coverage was accounted for in different age groups (Table S2, Supplementary Material), based on the 2016–2017 report of the National Influenza Immunization Coverage Survey in Canada [22]. Parameter values and their respective ranges are reported in Table 1.

**Table 1.** Parameters and their associated ranges used for simulating model scenarios

Parameter description	Baseline value	Range
*Transmissibility*	0.079 (calibrated)	Transmissibility was estimated by calibrating the model to the basic reproduction number *R*_0_ = 1.4
*Infection parameters*		
Latent period	1.5 days (mean)	1–2 days (uniform)
Infectious period	3.38 days (mean)	Log-normal distribution, scale = 1; shape = 0.4356
Probability of developing asymptomatic infection	0.5	0.3–0.7
Relative transmissibility of asymptomatic infection (compared to symptomatic infection)	0.5	Assumed
*Vaccine parameters*		
Efficacy	Varied in different scenarios	0.2–0.8 Corresponding to infection prevention in randomised control trials involving healthy individuals
Effectiveness	Varies, calculated based on the frailty index sampled for each individual	Between 0 and the assumed vaccine efficacy
*Avoidance behaviour*		
Susceptible individuals	Varied in different scenarios	0.4–0.8
Vaccinated individuals	Varied in different scenarios	Between 0 and the assumed parameter of avoidance behaviour for susceptible individuals

### Model implementation and simulations

We implemented the model in Julia language and performed Monte-Carlo simulations to analyse the outcomes as the average of sample realisations in different scenarios of disease spread. Individuals were vaccinated before the start of the epidemic, based on the given vaccine coverage in different age groups. During the simulations, the daily sampled contacts of a susceptible (vaccinated) individual were discarded with the probability *p*_s_ (*p*_v_), corresponding to the contact avoidance factor, if the contact was identified as a symptomatic infection. We ran simulations with varying vaccine efficacy and contact avoidance in their respective ranges, and compared the outcomes with the baseline scenario in which the epidemic unfolds without vaccination. All simulations were seeded with a randomly selected individual in the latent state of the disease, and averaged over 2000 independent realisations. We analysed the outputs to determine (ii) the relative epidemic size (compared to the scenario without vaccination); and (ii) per-contact risk-of-infection in different age groups.

## Results

### Contact avoidance

**Fig. 1.**
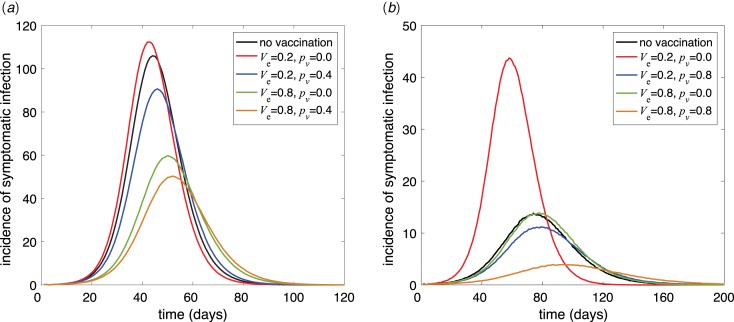
Incidence of symptomatic infection with (a) *p*_s_ = 0.4 and (b) *p*_s_ = 0.8 for different values of the vaccine efficacy and contact avoidance for vaccinated individuals.

For each simulated curve of epidemic, we collected data for the incidence of infection and the type of contacts in terms of disease states and age of individuals. We considered two scenarios of *p*_s_ = 0.4 and *p*_s_ = 0.8 as the parameters of contact avoidance by susceptible individuals. Figure 1(a, b) illustrates the average of symptomatic incidence for several values of avoidance *p*_v_. For a low vaccine efficacy (*V*_e_ = 0.2), the incidence of symptomatic infection grows faster with higher peak than the scenario without vaccination when vaccinated individuals practice no avoidance (*p*_v_ = 0). When the efficacy of vaccine is sufficiently high, or the avoidance of vaccinated individuals approaches that of susceptible individuals, the rate of disease spread reduces below that of the scenario without vaccination, with lower magnitude of disease incidence at the peak (Fig. S6, Supplementary Material).

### Epidemic size

**Fig. 2.**
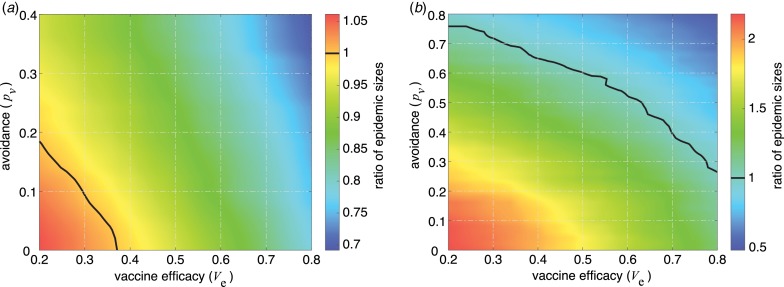
Ratio of epidemic size in the scenario with vaccination to the scenario without vaccination as a function of the vaccine efficacy and contact avoidance for vaccinated individuals. For given parameters *V*_e_ and *p*_v_, a ratio greater than one indicates that the total number of infections with vaccination is higher than that without vaccination. The avoidance behaviour for susceptible individuals is (a) *p*_s_ = 0.4 and (b) *p*_s_ = 0.8.

We calculated the epidemic size as a function of vaccine efficacy and contact avoidance. Figure 2(a,b) shows the ratio of epidemic size with vaccination to that obtained in the scenario without vaccination. The results indicate that there is a sizable domain of vaccine efficacy and contact avoidance by vaccinated individuals that leads to higher epidemic sizes (i.e. the region below the black curve of unity) compared to the no-vaccine scenario. We observed a larger domain for increased ratio of epidemic sizes with *p*_s_ = 0.8 (Fig. 2b) than with *p*_s_ = 0.4 (Fig. 2a). This is because higher levels of contact avoidance by susceptible individuals reduces the returns of vaccination, and therefore the impact of avoidance by vaccinated individuals becomes more pronounced in reducing the epidemic size.

**Fig. 3.**
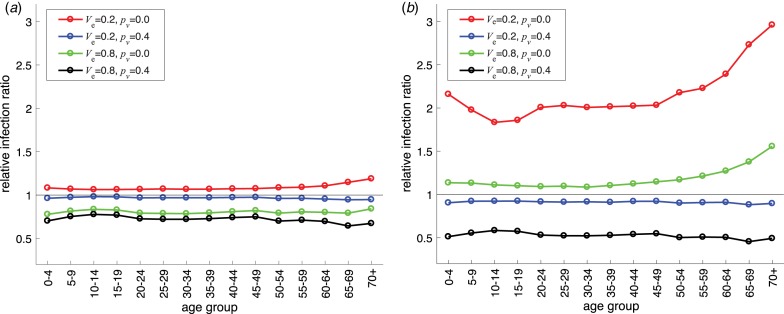
Age-specific relative infection ratio in the scenario with vaccination to the scenario without vaccination. A ratio greater than one indicates that the total number of infections in an age group with vaccination is higher than that in the same age group without vaccination. The avoidance behaviour for susceptible individuals is (a) *p*_s_ = 0.4 and (b) *p*_s_ = 0.8. Colour curves correspond to different values of vaccine efficacy and contact avoidance for vaccinated individuals.

We further analysed the simulation outcomes for the relative infection ratio in different age groups in specific scenarios. Figures 3 shows the ratio of total infections in vaccination scenarios to that in the absence of vaccination. When *p*_s_ = 0.4, Fig. 3a illustrates the possibility of a ratio greater than one for a low-efficacy vaccine (*V*_e_ = 0.2) if the avoidance level of *p*_v_ is less than *p*_s_ (red curve). We observed this possibility for sufficiently small *p*_v_ even with a high-efficacy vaccine (Fig. 3b, green curve). More importantly, when the ratio is greater than one, it increases for older age groups, suggesting that a larger number of infections may occur if vaccination causes contact avoidance to drop significantly among vaccinated individuals. When susceptible individuals practice a high level of avoidance *p*_s_ = 0.8, our results suggest that maintaining high avoidance among vaccinated individuals with a low-efficacy vaccine (Fig. 3b, blue curve) outperforms the scenario of a high-efficacy vaccine without avoidance (Fig. 3b, green curve).

Since avoidance is considered (and is in general practical) for contacts between susceptible and symptomatically infectious individuals, we also evaluated the contribution of both symptomatic and asymptomatic infections to the epidemic size. Not surprisingly, as the vaccine efficacy increases, the contribution of symptomatic infection to the cumulative incidence decreases, suggesting that a higher fraction of epidemic size is caused through contacts with asymptomatic infection (Fig. S7, Supplementary Material). However, we also observed the same pattern when vaccinated individuals increased their contact avoidance, with a steady decline in the fraction of total infections caused by symptomatic cases (Fig. S7, Supplementary Material). These findings show that the cumulative incidence attributed to contacts with asymptomatic infection increases as both vaccine efficacy and contact avoidance increase, while the epidemic size decreases.

### Risk-of-infection

**Fig. 4.**
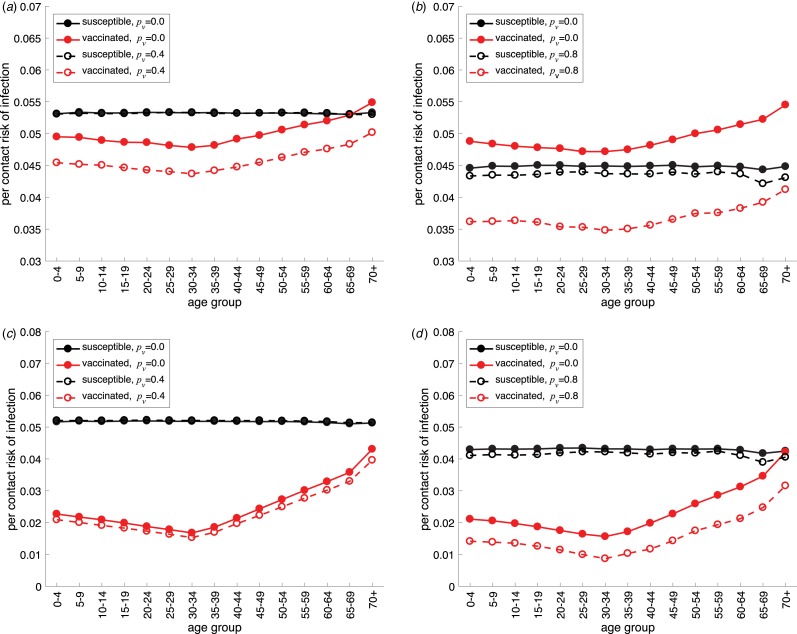
Per-contact risk-of-infection in different age groups in the presence of vaccination. Vaccine efficacy is (a, b) *V*_e_ = 0.2 and (c, d) *V*_e_ = 0.8. The parameter of contact avoidance for susceptible individuals is (a, c) *p*_s_ = 0.4 and (b, d) *p*_s_ = 0.8. Colour curves correspond to the risk-of-infection in susceptible individuals (black) and vaccinated individuals (red) with different avoidance parameter *p*_v_.

We evaluated the per-contact risk-of-infection in different age groups throughout the epidemic using simulation data for maximum likelihood estimators. For a low vaccine efficacy (*V*_e_ = 0.2), the risk-of-infection was highest in all age groups in the absence of contact avoidance by vaccinated individuals (Figs 4(a,b)). Importantly, when susceptible individuals practice a high level of contact avoidance, the per-contact risk-of-infection may be higher in vaccinated individuals with low levels of contact avoidance (Fig. 4b). Naturally, the lowest risk-of-infection was associated with the highest vaccine efficacy (*V*_e_ = 0.8) when vaccinated individuals practice the same level of contact avoidance as susceptible individuals (Figs 4(c,d)). We also observed that the risk-of-infection in vaccinated individuals increased for older age groups, especially in scenarios with low levels of contact avoidance (Fig. 4; red curves), which follows the pattern of the frailty index.

## Discussion

Based on a comprehensive agent-based simulation model, we found that a low vaccine efficacy can result in a higher number of infections (compared to the no-vaccine scenario) if vaccinated individuals reduced their level of engagement in behavioural avoidance below that of the susceptible individuals. Although vaccine-induced immunity decreases the risk-of-infection at the individual level, the larger epidemic size can still occur for a sufficiently low level of behavioural avoidance (within a feasible and non-negligible range of contact avoidance) even when the vaccine efficacy is high, with increasing number of infections in older age groups.

As we have shown, reduced levels of avoidance behaviour could potentially offset the benefits of vaccination in lowering the risk of contracting infection. Paradoxically, since influenza infection (if occurs) following vaccination is more likely to be subclinical [29, 30], behavioural avoidance becomes impractical and therefore a higher fraction of cumulative incidence may be caused by contacts with asymptomatic cases (Fig. S7, Supplementary Material). This has also been studied for the population-effects of suppressing clinical symptoms during influenza infection [31], which may lead to interaction among individuals with potential for disease transmission. In the context of imperfect vaccines, our modelling observations suggest that vaccination campaigns should pay particular attention to improving behavioural avoidance while highlighting the protective effects of vaccine-induced immunity.

Another important implication of our study relates to the target groups for vaccination. Most vaccination programmes for seasonal influenza prioritise specific groups in the population based on age (elderly people), health status and potential for severe disease outcomes (e.g. immunocompromised individuals and those with co-morbid illness), and exposure to infection (e.g. healthcare workers). A significant portion of target groups is subject to a high frailty index, which impairs the ability to resist influenza infection and respond to vaccination in a process known as immunosenescence [32–34]. Individuals with low frailty, including school children and healthy adults, are generally not listed in vaccine prioritisation. Importantly, these individuals contribute to a substantial portion of disease transmission due to their age-specific pattern and sheer volume of contacts [35, 36]. Inclusion of healthy children in vaccination programmes against influenza could blunt the level of transmission in the population. While quantitative evaluation of vaccinating children, based on data from countries that have implemented this strategy (e.g. England that prioritises all children aged 2–9 years [37]), is required to assess its population-wide benefits, our results suggest that higher vaccine effectiveness in healthy children and adults may have a large impact on reducing disease transmission.

This study is particularly relevant to the 2017–2018 influenza season with interim estimates of low vaccine effectiveness against A(H3N2) strain, reporting 17% in Canada [16], 10% in Australia [38] and 25% in the USA [14]. The public health implications of low vaccine effectiveness have been well recognised [15], especially for vaccines produced by the conventional egg-based technology that is prone to antigenic changes in egg-adapted strains [4, 39]. New vaccine technologies, including cell-based [15, 40], deoxyribonucleic acid (DNA)-based [41], and virus-like particle-based [42, 43], have shown to provide a promising path towards universal immunisation with a broader spectrum of immune responses [15]. While these technologies aim to improve vaccine effectiveness, they also have the advantage of short-timelines over egg-based technology for the rapid and large-scale production of strain-specific vaccines that can be used to mitigate the impact of emerging influenza viruses with pandemic potential. However, despite the low effectiveness conferred by egg-based vaccines, influenza vaccination remains an important public health measure to reduce the severity of disease and its associated costs, especially in the geriatric population [29, 30].

Our findings in this study are based on Monte-Carlo stochastic simulations, taking into account heterogeneities of contacts and variability in vaccine effectiveness at the individual level. We parameterised the model with Canadian databases for population demographics, vaccination coverage and the age-dependent frailty index that affected vaccine effectiveness. However, we note a number of limitations that arise from the lack of data and evidence. For example, the baseline transmission probability in the process of Bernoulli trial was assumed to be the same in all contacts regardless of the duration of each contact. In many communicable diseases, including influenza, disease transmission is affected by close-range contacts between individuals and the time spent in contact [18], and therefore different contact durations might yield very different transmission probabilities [18, 44, 45]. Some contacts are very short and correspond to a small transmission probability, but many contacts are long and could play a crucial role in disease dynamics. While we have simulated the model for a specific reproduction number, it is worth noting that the qualitative aspects of our results are independent of the choice of *R*_0_, which is adjusted in the transmission probability during the calibration process. As disease transmission depends on clinical status of an infectious individual, we relied on previous studies and assumed that asymptomatic infection is 50% less infectious than symptomatic infection [27]. Although viral shedding in patients with asymptomatic influenza occurs [46], the evidence for the level of disease transmissibility from asymptomatic infection is weak [47]. In the model presented here, we did not consider the effect of pre-existing (or cross-reactive) immunity due to prior vaccination or natural infection. The duration and strength of such immunity can influence both the epidemic size and effectiveness of vaccination [48, 49], but this seems likely to be balanced in our calculation by the possibility of developing asymptomatic infection in 30%–70% of susceptible individuals who became infected. Despite these limitations that merit further consideration as more data and evidence become available, our results indicate an important interplay between vaccine effectiveness and behavioural avoidance in disease dynamics. Beyond theoretical investigation [10, 12], quantitative studies are needed to estimate the impact of behavioural responses in the context of vaccination during seasonal influenza epidemics.

## Supplementary material

For supplementary material accompanying this paper visit https://doi.org/10.1017/S0950268818003540. click here to view supplementary material

## Data

The computational system and data for reproducibility is available at https://github.com/thomasvilches/Influenza2.
