# Supplementary material for: Risk of influenza infection with low vaccine effectiveness: the role of avoidance behaviour
Source: Epidemiol Infect. 2019 Jan 25;147:e75. doi: 10.1017/S0950268818003540 (PMC6518843; doi:10.1017/S0950268818003540)
Supplement: Supplementary file 1 [file S0950268818003540sup001.docx]

**Supplementary Material**

**Risk of Influenza Infection with Low Vaccine Effectiveness: the Role of Avoidance Behaviour**

**Thomas N. Vilches,^1^ Majid Jaberi-Douraki,^2^ Seyed M. Moghadas^3^**

^1^Institute of Biosciences, Department of Biostatistics, São Paulo State University, Botucatu SP 18618-689, Brazil

^2^Institute of Computational Comparative Medicine, Department of Mathematics, and Department of Anatomy and Physiology, Kansas State University, Manhattan, KS 66506, USA

^3^Agent-Based modelling Laboratory, York University, Toronto, Ontario M3J 1P3, Canada

This supplemental provides further details of model structure and analysis of pertinent data for its parameterization.

**Model calibration**

The model was calibrated by iteratively varying the baseline transmission probability, so that the average of sample realizations reached the assumed $R_{0}=1.4$. By definition, $R_{0}$ represents “the average number of secondary infections that the first infectious individual generates in an entirely susceptible population”. Therefore, for each simulation, we counted the number of symptomatic infections (which represents the estimates for $R_{0}$) caused by the first infectious case seeded into the population, during the infectious period sampled for this case. The calibration was performed in the absence of any control measure (i.e., vaccination or contact avoidance).

**Population demographics and contact patterns**

The model simulates the spread of influenza in an age-stratified population with 10,000 individuals. The population age distribution was derived from the 2017 Canadian census data^[[1]](#endnote-1)^, as presented in Figure S1. After selecting for the age groups, the age of each individual was sampled uniformly from the associated range. We assigned a number of daily contacts to each individual, which was sampled (for each day) from a negative-binomial distribution^[[2]](#endnote-2)^, with age-dependent mean and standard deviation given in Table S1. Simulated daily number of contacts for each age group in the model is illustrated (in log-scale) in Figure S2.

**Figure S1 –** Age distribution of the population (Canadian census data, 2017).

**Table S1 –** Mean and standard deviation for the daily number of contacts in different age group.

| Age | Mean (standard deviation) of number of daily contacts |
| --- | --- |
| 0-4 | 10.21 (7.65) |
| 5-9 | 14.81 (10.09) |
| 10-14 | 18.22 (12.27) |
| 15-19 | 17.58 (12.03) |
| 20-29 | 13.57 (10.60) |
| 30-39 | 14.14 (10.15) |
| 40-49 | 13.83 (10.86) |
| 50-59 | 12.30 (10.23) |
| 60-69 | 9.21 (7.96) |
| 70+ | 6.89 (5.83) |

**Figure S2 –** Simulated daily number of contacts between age groups represented in log-scale.

**Infectious period distribution**

The infectious period of an infected individual was sampled from a truncated lognormal distribution, with the mean of 3.38 days (and maximum length of 15 days), as illustrated in Figure S3.

**Figure S3.** Lognormal distribution of the infectious period.

**Vaccination**

We included behavioural responses of individuals in the presence of vaccination. We assumed that vaccinated individuals are less likely to practice avoidance behaviour compared to unvaccinated individuals in terms of avoiding their contacts with symptomatically infectious individuals. Although there is a vast literature on the perception of risks and benefits prior to receiving a vaccine [Refs], there is no study to quantify the risk-of-infection perception following vaccination. However, it is reasonable to assume that vaccinated individuals may perceive their risk-of-infection reduced due to protective effects of the vaccine, and therefore, may practice lower level of precaution in avoiding potentially infectious contacts. We therefore considered the parameter of avoidance behaviour for vaccinated individuals to be less than that of susceptible individuals and may reach the same level at its maximum.

Vaccination was implemented as pre-epidemic strategy, and coverage was achieved for different age groups in random vaccination^[[3]](#endnote-3)^. Table S2 summarizes the vaccine coverage for different age groups reported in the National Influenza Immunization Coverage Survey, Canada, 2016-2017.

**Table S2.** Vaccine coverage for aggregated age groups.

| Age | Coverage (95% confident interval) |
| --- | --- |
| 6 months – 4 years | 26.5 (20.1–32.9) |
| 5 – 12 years | 23.0 (18.5–27.4) |
| 13 – 17 years | 23.2 (17.0–29.4) |
| 18 – 49 years | 22.7 (19.4–25.9) |
| 50 – 64 years | 38.2 (34.2–42.3) |
| 65+ | 69.5 (65.5–73.4) |

**Frailty index and vaccine effectiveness**

Frailty index data of the 2014 Canadian Community Health Survey of chronic diseases were fit using segmented linear regression analysis^[[4]](#endnote-4)^. We considered the frailty index $F\left( a \right)$ as a function of age in the fitting process, and sampled for each individual of age ‘$a$’ from a uniform distribution with the mean of $F\left( a \right)$ in the range $F\left( a \right)\pm0.05$.

**Figure S4.** Piecewise linear regression was fitted to the frailty index data (circles) as a function of age.

Using the sampled frailty index, we calculated individual-level vaccine effectiveness (the level of protection against infection) and averaged over each age group. Figure S5 illustrates vaccine effectiveness in different age groups, based on given vaccine efficacies ($V_{\text{e}}$).

**Figure S5.** Mean vaccine effectiveness in different age groups.

**Incidence of symptomatic infection**

We recorded the daily number of new infections who developed symptoms in each simulation for different scenarios of vaccine efficacy and contact avoidance. Figure S6 represents the simulation curves (blue curves) and their average (red curve) during the epidemic.

**Figure S6.** Incidence of symptomatic infection for the scenarios of $p_{s}=0.4$ (A1-A5) and $p_{s}=0.8$ (B1-B5). Simulations were run for 2000 independent realization (blue curves) to determine their average (red curves) in the absence of vaccination (A1, B1); and in the presence of vaccination with $V_{e}=0.2,p_{v}=0$ (A2); $V_{e}=0.2,p_{v}=0.4$ (A3); $V_{e}=0.8,p_{v}=0$ (A4); $V_{e}=0.8,p_{v}=0.4$ (A5); $V_{e}=0.2,p_{v}=0$ (B2); $V_{e}=0.2,p_{v}=0.8$ (B3); $V_{e}=0.8,p_{v}=0$ (B4); $V_{e}=0.8,p_{v}=0.8$ (B5).

**Relative contributions of symptomatic and asymptomatic infections to the epidemic size**

In each simulation, we recorded the type of contacts between susceptible and infectious individuals to determine the relative contributions of symptomatic and asymptomatic infections to the cumulative incidence throughout the epidemic. Figure S7 shows the proportion of infections that were cause by symptomatic (A, C) and asymptomatic (B, D) infections for different values of vaccine efficacy, when the contact avoidance of susceptible individuals ($p_{s}$) is 0.4 (A, B) and 0.8 (C, D), and the contact avoidance of vaccinated individuals varies in the rage represented on the *x*-axis. As evident, contact avoidance decreases the number of infections caused by symptomatic cases; however, the fraction of new infections caused by asymptomatic infections increases. This is expected as contact avoidance is implemented for possible contact between susceptible and symptomatically infectious individuals. Furthermore, since contact avoidance with asymptomatic infections is impractical, contacts can lead to disease transmission, which may increase the epidemic size.

**Figure S7.** Fraction of total infections caused by symptomatic and asymptomatic cases as a function of $p_{v}$ for different vaccine efficacy, with $p_{s}=0.4$ (A,B); and $p_{s}=0.8$ (C,D).

**Average number of cases per index**

We calculated the average number of cases that is generated by an infectious individual in different age groups in the absence of vaccination. For instance, an infectious individual in the age group 20-24 produced, on average, one new infection, both for $p_{s}=0.4$ (black curve) and $p_{s}=0.8$ (red curve). This result is clearly related to and follows the behaviour of the average number of daily contacts per age group, presented in Table S1.

**Figure S8.** Average number of new cases generated by an infected individual in an age group in the absence of vaccination with $p_{s}=0.4$ (black curve) and $p_{s}=0.8$ (red curve).

**Age-dependent number of cases per index**

We used the total number of cases in each age group and their contact information recorded in simulations to derive the average number of cases in an age group generated by an infectious case in any other age group. In the absence of vaccination, Figure S9 represents the results for $p_{s}=0.4$ (A) and $p_{s}=0.8$ (B).

**Figure S9.** Average number of new cases generated by an infected individual in different age groups in the absence of vaccination with $p_{s}=0.4$ (A) and $p_{s}=0.8$ (B).

**References**

1. . Website of Government of Canada at http://www5.statcan.gc.ca/cansim/a26?lang=eng&retrLang=eng&id=0510001&&pattern=&stByVal=1&p1=1&p2=37&tabMode=dataTable&csid=. Accessed February 2018. [↑](#endnote-ref-1)
2. . Mossong, J., Hens, N., Jit, M., Beutels, P., Auranen, K., Mikolajczyk, R., Massari, M., Salmaso, S., Tomba, G.S., Wallinga, J., Heijne, J., Sadkowska-Todys, M., Rosinska, M., Edmunds, W.J., 2008. Social contacts and mixing patterns relevant to the spread of infectious diseases. PLoS Med. 5, 0381–0391. https://doi.org/10.1371/journal.pmed.0050074 [↑](#endnote-ref-2)
3. . Public Health Agency of Canada, 2018. 2016/17 Seasonal Influenza Vaccine Coverage in Canada. [↑](#endnote-ref-3)
4. . Government of Canada’s website available at http://infobase.phac-aspc.gc.ca:9600/PHAC/dimensionMembers.jsp?l=en&rep=i3212B12F133F4CE88AD13DB60CA37237&s#. Accessed March 2018. [↑](#endnote-ref-4)
